# NRG1 and KITL signal downstream of retinoic acid in the germline to support soma-free syncytial growth of differentiating spermatogonia

**DOI:** 10.1038/cddiscovery.2015.18

**Published:** 2015-10-05

**Authors:** KM Chapman, GA Medrano, J Chaudhary, FK Hamra

**Affiliations:** 1 Department of Pharmacology, University of Texas Southwestern Medical Center, 6001 Forest Park Road, Dallas, TX 75390, USA; 2 Cecil H. and Ida Green Center for Reproductive Biology Sciences, University of Texas Southwestern Medical Center, 6001 Forest Park Road, Dallas, TX 75390, USA

## Abstract

Defined culture systems supporting spermatogonial differentiation will provide experimental platforms to study spermatogenesis. However, germline-intrinsic signaling mechanisms sufficient to support spermatogonial differentiation without somatic cells remain largely undefined. Here we analyzed EGF superfamily receptor and ligand diversity in rat testis cells and delineated germline-intrinsic signaling via an ERBB3 co-transducer, ERBB2, as essential for retinoic acid-induced syncytial growth by differentiating spermatogonia. Similar to the ERBB2/3 agonist NRG1, we found that KIT Ligand (KITL) robustly supported spermatogonial differentiation without serum or somatic cells. ERBB2 inhibitors failed to disrupt KITL-dependent spermatogonial development, and KITL prevented ERBB3-deficient spermatogonial degeneration upon differentiation. Thus we report that NRG1 and KITL activate alternative pathways downstream of retinoic acid signaling in the germline that are essential for stem cells to undergo premeiotic steps of spermatogenesis in culture. Robust serum/soma-free spermatogonial differentiation opens new doors to study mammalian germ cell biology in culture and to discover factors that can drive meiotic progression *in vitro*.

## Introduction

Spermatozoan development is maintained within the testes’ seminiferous epithelium by the process of spermatogenesis.^1^ Spermatogonial stem cells maintain spermatogenesis by their unique abilities to self-renew or produce spermatogonial syncytia that differentiate through meiosis to form haploid gametes termed round spermatids.^2^ Round spermatids undergo a complex metamorphosis to develop into fully elongated spermatozoa by the process of spermiogenesis.^1^ Chemically defined culture systems that support spermatocyte maturation and spermatid development from mammalian spermatogonial stem cell lines remain to be established but, prospectively, will allow researchers to probe diverse premeiotic, meiotic and postmeiotic germ cell processes.^1^

The ability to study spermatogenesis *in vitro* is being held at bay because culture systems that robustly support spermatogonial proliferation and/or differentiation into meiosis do not exist for most mammalian species outside rodents. In rodents, donor spermatogonial stem cells can be maintained long term in culture^3^ but can only be cultured through meiosis in recipient testes^4^ or in organ culture within seminiferous tubules.^5^ Going forward, fully defined culture systems that effectively support spermatid production from spermatogonial stem cell lines will need to be established from diverse Animalia to realize the full potential of *in vitro* spermatogenesis for experimentally dissecting cellular processes and for producing haploid gametes.

‘A-single (A_s_)’ spermatogonia function as spermatogonial stem cells that initiate spermatogenesis during development into syncytia containing 2–32 ‘undifferentiated’ A-paired (A_pr_) and A-aligned (A_al_) progenitor spermatogonia.^6,7^ In rodents, undifferentiated spermatogonia mitotically arrest during seminiferous epithelial cycle stages VI–VIII and then transform into ‘differentiating’ type A1 spermatogonia under control of KITL and retinoic acid (active vitamin A derivative).^8,9^ Type A1 spermatogonia re-enter the mitotic cell cycle and give rise to subsequent generations of differentiating spermatogonia (types A2>A3>A4>Int>B),^10^ by which time germ cell numbers/syncytium can be amplified >100-fold prior to entering meiosis to form spermatocytes.^11^

Polypeptides encoded by *glial cell line derived neurotrophic factor* (*Gdnf*) and *fibroblast growth factor* (*Fgf*) are essential for mouse spermatogonial stem cells to maintain spermatogenesis.^12,13^ GDNF and FGF2 also support proliferation of mouse,^14,15^ rat^16,17^ and hamster^18^ spermatogonial stem cells in culture. In fact, GDNF and FGF2 are essential components in serum-free media that maintain mouse spermatogonial stem cell proliferation long term without somatic cells.^19^ WNT-family polypeptides accelerated mouse spermatogonial stem cell proliferation in a serum-free medium containing GDNF, GFRa1 and FGF2 but did so indirectly by signaling in early progenitor spermatogonia.^20,21^ In contrast to highly defined culture systems that maintain spermatogonial stem cell proliferation, a large knowledge gap exists on signaling pathways that can act directly in germ cells to support additional premeiotic, meiotic or postmeiotic spermatogenic differentiation steps independent of a somatic environment.

KITL is a prominent Sertoli cell-derived polypeptide growth factor essential for differentiating spermatogonia development *in vivo*.^8^ Signaling via the KITL receptor KIT is also essential for differentiating spermatogonia development *in vivo*.^22,23^ KIT has been reported on differentiating spermatogonia, early spermatocytes and Leydig cells.^24,25^ Retinoic acid is a vitamin A-derived steroid hormone and is known for its ability to regulate spermatogenic cell differentiation in testes,^26^ organ cultures,^27,28^ isolated testis cell cultures,^29^ cultures enriched with prospermatogonia^30^ and mouse spermatogonial lines.^31^ Still, to date, neither KITL nor retinoic acid have been defined as essential to support robust clonal development/syncytial growth of differentiating spermatogenic cells without somatic cells.

One germline receptor, ERBB3, was recently genetically annotated as essential for syncytial growth of differentiating spermatogenic cells in a serum/soma-free medium containing NRG1, GDNF, FGF2 and retinoic acid (i.e., SD Medium).^32^ However, *Erbb3*-null germlines fully supported spermatogenesis in recipient rat testes, indicating that additional spermatogonial growth factors existed in rats.^32^ Moreover, NRG1 is an ERBB3-ligand, but Sertoli cell-derived *Nrg1* was specifically required for meiosis in mice.^33^ Here, by in-depth analysis of EGF-family signaling molecules expressed in rat spermatogenic cells and growth factor components in SD Medium, we have defined alternate ERBB2-dependent and ERBB2-independent growth factor signaling pathways that act directly in the rat germline with retinoic acid to robustly support syncytial growth of differentiating spermatogonia without somatic cells.

## Results

### ERBB2 and ERBB3 are selectively detected in rat spermatogonia

**Figure 1:**
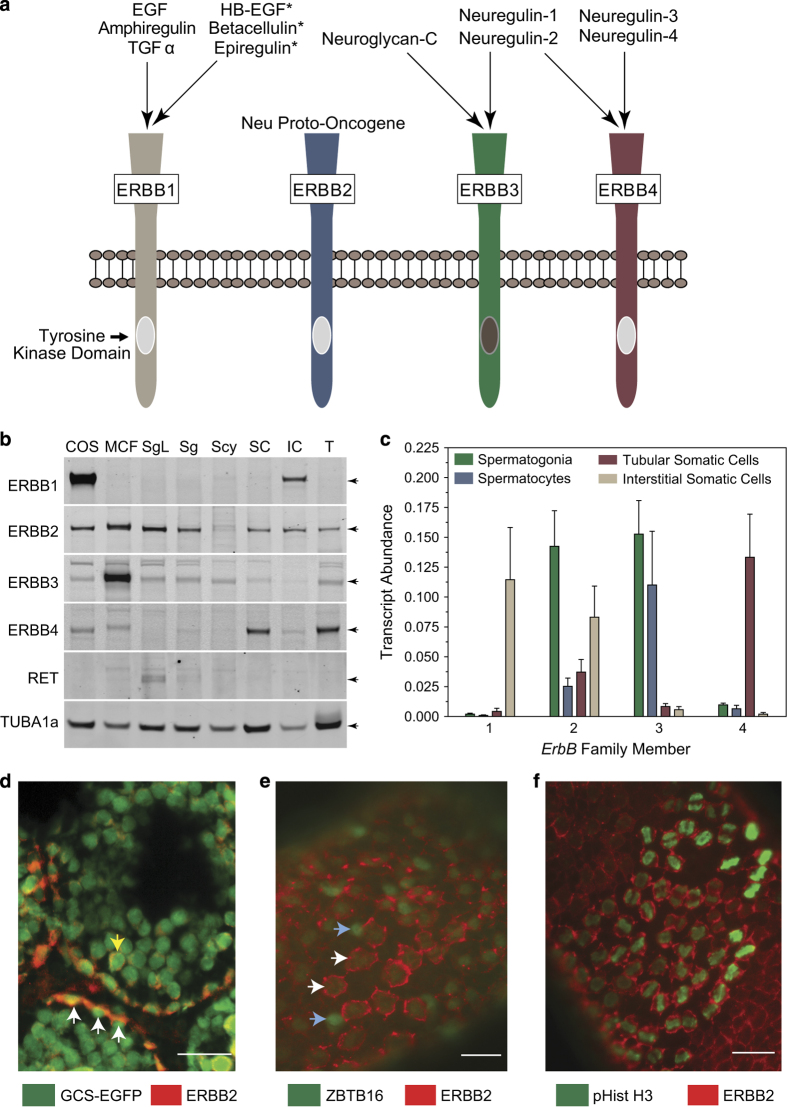
ERBB-family signaling molecules in rat testis cells. (**a**) Polypeptides in the EGF super-family signal by activating ERBB-family transmembrane receptor tyrosine kinases. ERBB1 is a receptor for ‘classical’ low molecular weight EGF-like peptides. ERBB2 is the primary transducer for ligand-bound ERBB1, ERBB3 and ERBB4. ERBB2’s extracellular domain does not bind known ligands. ERBB3 is a receptor for Neuregulin-1 (NRG1), NRG2 and Neuroglycan-C (CSPG5). Ligand bound ERBB3 displays poor kinase activity and signals most effectively as a heteromer with ERBB1, ERBB2 and/or ERBB4. ERBB4 is a receptor for NRG1, NRG2, NRG3 and NRG4 plus other EGF-like peptides*. (**b**) Western blotting analysis of ERBB-family proteins in fractions of testis cells from 23-day-old rats. Lysates of type A spermatogonia after proliferating for ~180 days/15 passages in culture (SgL), freshly isolated laminin-binding type A spermatogonia (Sg), laminin non-binding spermatogenic cells (Scy), tubular somatic cells (SC), interstitial somatic cells (IC), MCF7 human mammary gland cells (MCF) and COS7 monkey kidney cells (COS). Arrowheads: ERBBs 1–4 (~185 kDa), RET (~155 and 170 kDa) and TUBA1a (~55 kDa). (**c**) Relative abundance (qtPCR) of ERBB-family transcripts in testis cells isolated from 23-day-old rats (*n*=cells from three different rats; ±S.E.M.). Spermatogonia (Sg), Spermatocytes (Scy; differentiating spermatogonia/early spermatocytes), Tubular somatic cells (SC) and Interstitial somatic cells (IC) are cell types described in panel (**b**). (**d**) Testis cross-section from 26-day-old tg*GCS-EGFP* transgenic rats labeled with anti-ERBB2 (Red) overlaying EGFP fluorescence from germ cells (green). Note, cytoplasmic ERBB2 labeling in germ cells resembling differentiating spermatogonia (white arrows) and spermatocytes (yellow arrow). Scale, 40 *μ*m. (**e**) Rat seminiferous tubule whole mount from 24-day-old wild-type rat labeled using antibodies to ERBB2 (Red) and ZBTB16 (Green). Scale, 20 *μ*m. Note: nuclear ZBTB16 labeling is more robust in ERBB2-dim spermatogonia (cyan arrows), compared with ERBB2-bright spermatogenic cells (white arrows). (**f**) Rat seminiferous tubule whole mount from a 24-day-old wild-type rat labeled with antibodies to ERBB2 (Red) and phospho-Histone-3 (pH3, Green). Scale, 40 *μ*m. Note: nuclear pH3 in large mitotic ERBB2^+^ syncytia.

ERBB3 (HER3 in humans) is encoded by one of four different mammalian *erythoblastoma virus B homolog* genes (i.e., *Erbb1*,* Erbb2*,* Erbb3*, *Erbb4*) (Figure 1a).^34^ In somatic tissues, ligand-bound ERBB3 signals as an obligate heteromer with other ERBB-family transmembrane receptor tyrosine kinases.^34^ To identify potential signaling partners for ERBB3 in the germline, we analyzed ERBB-family proteins in isolated rat spermatogenic and somatic testis cells by western blotting. ERBB2 and ERBB3 (~185 kDa bands) were detected in freshly isolated undifferentiated type A spermatogonia and in a rat spermatogonial stem cell line enriched for the GDNF/GFRa1 signal transducer, RET (Figure 1b).

ERBB3 was detected at similar levels in differentiating spermatogonia/early spermatocyte preparations compared with the less differentiated type A spermatogonia (Figure 1b). However, ERBB2 abundance was reduced in differentiating spermatogonia/early spermatocytes relative to type A spermatogonia (Figure 1b). Compared with ERBB2 and ERBB3, ERBB1 and ERBB4 were most abundant in interstitial and tubular somatic cells, respectively (Figure 1b). A relatively weak ERBB4 band was detected in freshly isolated type A spermatogonia, but not in the rat spermatogonial line (Figure 1b). *Erbb*-family transcript profiles generated by qtPCR and RT-PCR (Figure 1c, Supplementary Figure S1a) correlated with the relative abundance of ERBB-family proteins in testis cells by western blotting (Figure 1b). Thus ERBB2 and ERBB3 were the predominant ERBB-family members detected within rat spermatogenic cells.

We previously detected ERBB3 by antibody labeling selectively on a novel sub-population of type A single spermatogonia in rats.^35^ Because ERBB2 was detected as a potential signaling partner for ERBB3 in spermatogonia (Figure 1b), we analyzed ERBB2 localization in sections and whole mounts of rat seminiferous tubules. ERBB2 labeling was detected on spermatogenic cells (tg*GCS*-*EGFP* ^+^) (Figure 1d) and selectively in differentiating type A spermatogonia (Figure 1e; white arrows, ZBTB16^lo^). ERBB2 localized to spermatogonial cytoplasm and was enriched at the plasma membrane of larger, synchronously dividing syncytia characteristic of differentiating spermatogonia (Figure 1f). Weaker ERBB2 labeling intensities were detected in spermatocytes (Figure 1d; yellow arrow) and undifferentiated type A spermatogonia (Figure 1e; cyan arrows; ZBTB16^hi^). Detection of ERBB2 on differentiating type A spermatogonia provided a candidate co-transducer for ERBB3-dependent development of differentiating rat spermatogenic cells in serum/soma-free cultures.^32^

### Rat testes express an array of EGF-family ligand mRNAs

**Figure 2:**
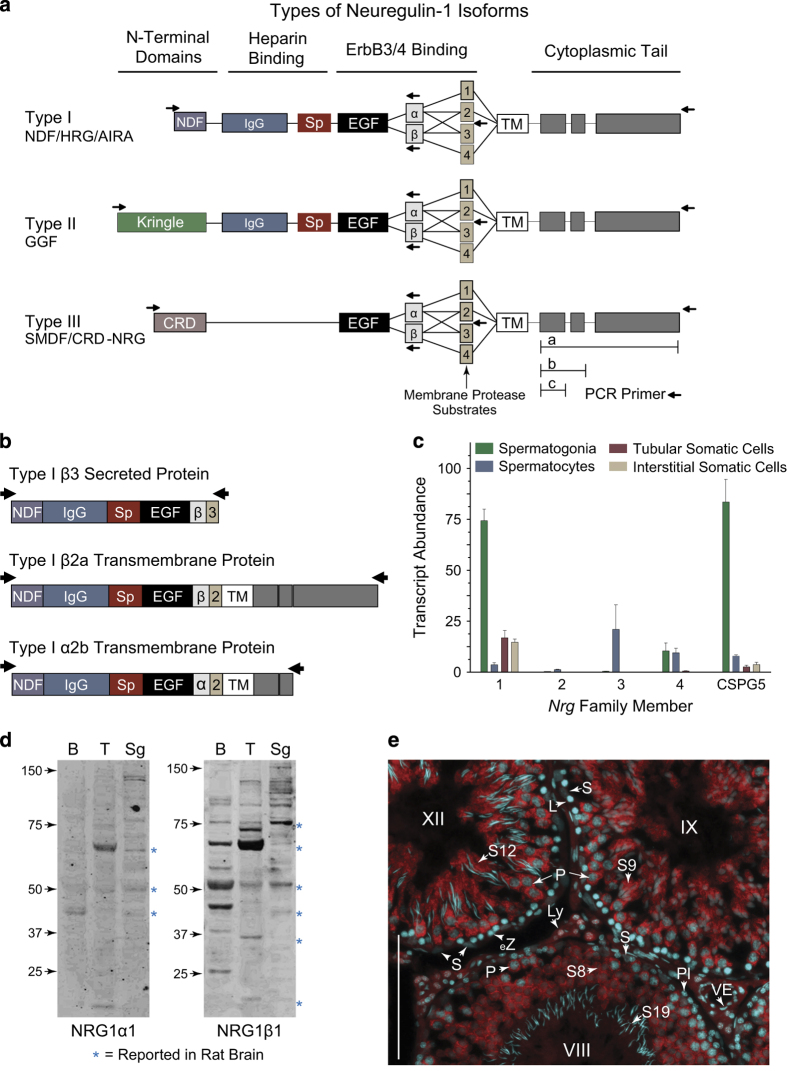
Spermatogenic cells selectively express neuregulin-family genes. (**a**) Strategy to analyze *Neuregulin-1* (*Nrg1*) mRNA splice variants. Exons (boxes) in Types I, II and III *Nrg1*. Distinct NRG1 Types are generated by alternatively splicing N-terminal exons (NDF, Kringle and CRD domains). IgG and Sp exons encode heavily glycosylated domains, which bind heparin sulfate proteoglycans.^58^ EGF homology domains (black boxes) together with either *α* or *β* domains (gray boxes) bind with high affinity to ERBB3 and ERBB4 extracellular domains. Exons designated by tan boxes 1–4 encode extracellular ‘stalk domains’, immediately upstream from the transmembrane domain (TM). Stalk domains 1, 2 and 4 are substrates for distinct metalloproteases, which regulate NRG1 extracellular domain shedding.^59^ NRG1’s with stalk domain 3 contain a C-terminal stop codon before the TM and are secreted. Exons in the gray boxes encoding different cytoplasmic domains that also regulate extracellular domain shedding. Arrows: PCR primers used to analyze testicular *Nrg1* variants. (**b**) Full-length *Nrg1* transcripts amplified from undifferentiated type A spermatogonia encode variants of Type I, NRG1. Arrows: respective PCR primers used to clone *Nrg1* variants. (**c**) Spermatogonia selectively express mRNAs encoding NRG1 and CSPG5 (qtPCR), *n*=cells from three different rats; ±S.E.M. Spermatogonia, Spermatocytes (differentiating spermatogonia/early spermatocytes), Tubular somatic cells and Interstitial somatic cells refer to Sg, Scy, IC and SC described in Figure 1b. (**d**) Western blots for NRG1*α*1 and NRG1*β*1 in the rat brain (B) and testis (T) and a primary spermatogonial line derived from undifferentiated type A spermatogonia (Sg). Arrows on left of each blot represent respective size molecular markers (kDa). Blue asterisks denote respective size NRG1 variants previously reported in the rat brain.^36–38^ (**e**) Immunolabeling for NRG1*α*1 (red cytoplasm) in adult rat testis sections. Nuclei counterstained with Hoechst 33342 dye (cyan). Pl=preleptotene spermatocyte; L=leptotene spermatocyte; eZ=early zygotene spermatocyte; P=mid-to-late pachytene spermatocytes; S8, S9, S12, S19=Step 8, 9, 12, 19 spermatids; VE=vascular endothelial cell; L=Leydig cell; S=Sertoli cell. Roman numerals denote spermatogenic stages. Scale bar, 100 *μ*m.

To analyze potential ligands for ERBB-family receptors in testes, we first surveyed transcripts encoding NRG1 variants expressed by germline and somatic testis cells (Figure 2a). *Nrg1* was selectively detected in rat type A spermatogonia by RT-PCR (Supplementary Figure S1b). Full open reading frames encoding secreted (Type 1*β*3) and transmembrane (Types 1*β*2a and 1*α*2b) *Nrg1* mRNA variants were cloned from type A spermatogonia (Figure 2b, Supplementary Figure S1c).

Similar to *Nrg1*, transcripts encoding other neuregulins, including *Ngr2*, *Ngr3* and *Csgp5* were selectively detected in spermatogenic cells (Figure 2c, Supplementary Figure S1b). *Nrg1* and *Csgp5* were most abundant in type A spermatogonia (Figure 2c, Supplementary Figure S1b), whereas *Nrg3* was most abundant in differentiating spermatogonia/early spermatocyte fractions (Figure 2c, Supplementary Figure S1b). In addition to transcripts encoding Neuregulins, spermatogenic cells expressed transcripts encoding multiple ligands for ERBB1 (*Egf*, *Tgfa*, *Areg*, *Btc*) and ERBB4 (*Btc*, *Hbefg*) (Supplementary Figure S1b).

Western blotting for ERBB3 agonists in rat testis and undifferentiated type A spermatogonia identified NRG1*α*1 and NRG1*β*1 variants/isoforms previously reported in the rat brain (Figure 2d, cyan asterisks).^36–38^ NRG1*α*1 and NRG1*β*1 ~65 kDa forms were most abundant in total testis; however, NRG1*β*1 ~75 and ~50 kDa forms were enriched in undifferentiated spermatogonia (Figure 2d). Antibody labeling for NRG1*α*1 and NRG1*β*1 variants in adult rat testis sections clearly detected cytoplasmic NRG1*α*1 in spermatogenic cells ranging from mid-late pachytene spermatocytes to step 17 elongating spermatids and also in Leydig cells (Figure 2e, Supplementary Figure S1d). NRG1*α*1 labeling was near background levels in spermatogonia, pre-leptotene to early pachytene spermatocytes and Sertoli cells (Figure 2e, Supplementary Figure S1d). NRG1*β*1 labeling was the strongest in late pachytene spermatocyte cytoplasm and Sertoli cell cytoplasm but less intense to background levels in other testis cell types (Supplementary Figure S1d). Thus rat testis cells collectively expressed transcripts encoding diverse EGF-family ligands and receptors, whereas rat type A spermatogonia selectively expressed gene products linked to activation of ERBB2 as a signal transducer for NRG1 and ERBB3 (Figure 2, Supplementary Figures S1b–d).

### ERBB2 inhibitors disrupt spermatogonial differentiation in a serum-free medium

**Figure 3:**
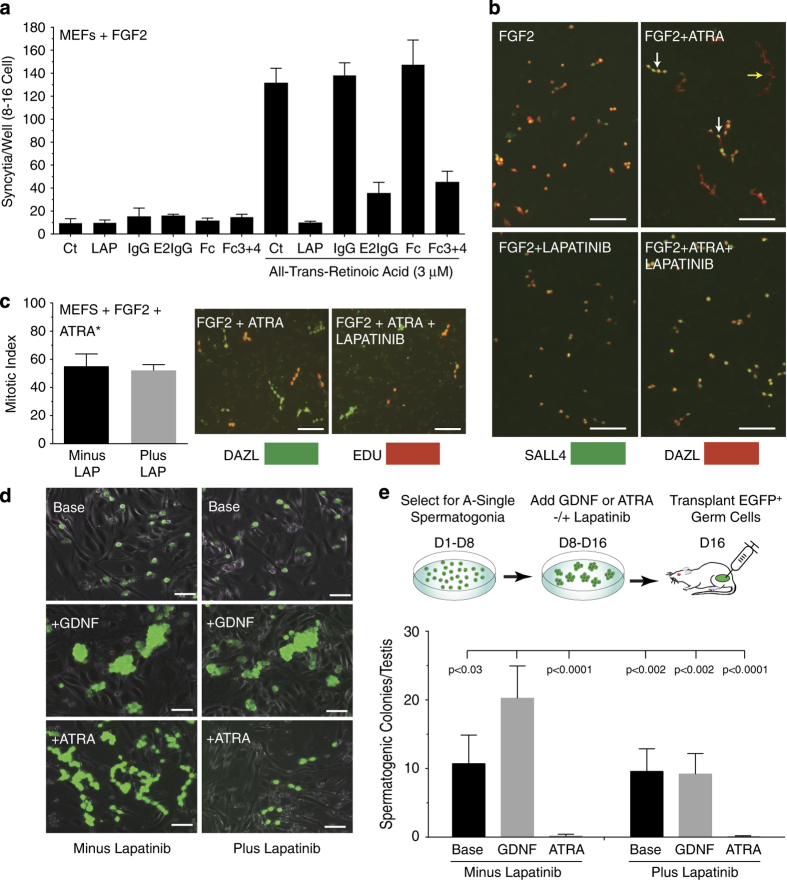
ERBB2 inhibitors block syncytial growth of spermatogenic cells. (**a**) ERBB2 inhibitors selectively block development of rat spermatogonia syncytia on MEFs in response to 3 *μ*M all-trans retinoic acid (ATRA). Lapatinib (1 *μ*M), Fc (6 *μ*g/ml), FcERBB3+FcERBB4 (3 *μ*g/ml+3 *μ*g/ml), Goat IgG (6 *μ*g/ml) and Goat ERBB2-neutralizing IgG (6 *μ*g/ml). Representative of duplicate colony forming assays (±S.E.M., triplicate wells); culture conditions described in panel (**b**). Scale, 100 *μ*m. (**b**) Rat spermatogonial line maintained on MEFs in SG^F^ Medium for 1 week and then for 6 additional days in SG^F^ Medium containing ATRA and/or Lapatinib, as in panel (**a**). Cells were labeled with antibodies to DAZL (Red) and SALL4 (Green) prior to scoring spermatogenic units. (**c**) Left, ERBB2 inhibition does not attenuate EDU incorporation by spermatogonia. Right, Spermatogonial cultures were passaged into SG^F^ Medium and maintained for 48 h before treating with retinoic acid for 4 days and then pulsing with EDU (red) for 7.5 h pior to fixing and antibody labeling for DAZL (green). Scale, 100 *μ*m. *Note: shorter incubation times in SG^F^ and SD media enabled effects of Lapatinib on EDU incorporation to be captured in longer syncitia prior to their degeneration upon differentiation in response to ATRA. (**d**) Images of a rat spermatogonial line after culture on MEFs in SG^F^ Medium for 7 days (D1–D8) and then in SG^F^ Medium supplemented with either GDNF or ATRA for 8 days (D8-D16). Note: Lapatinib selectively blocked survival of differentiating spermatogonia in response to ATRA. Scale, 50 *μ*m. (**e**) (Top) Diagram of culture experiment shown in panel (**a**), prior to transplanting respective spermatogonial cultures into recipient rat testes (*n*=4–6 rats/condition with right testis transplanted; *P*-values, multiple *t*-tests). (Bottom) Relative numbers of spermatogenic colonies generated/testis/1000 donor germ cells (tg*GCS-EGFP*^+^) harvested from cultures described in panel (**d**). Recipients analyzed 28 days posttransplantation.

Because ERBB2 was identified as a candidate NRG1/ERBB3 co-transducer in spermatogonia (Figures 1b–f), we tested the ability of highly selective ERBB2 inhibitors to block clonal development of differentiating spermatogenic cells *in vitro* on feeder layers of mouse embryonic fibroblasts (MEFs) (Figure 3). Rat spermatogonial stem cells were passaged and maintained for 1 week in spermatogonial culture medium (SG Medium) containing FGF2 but without GDNF (SG^F^ Medium; Table 1) to increase the percentage of A_s_ and A_pr_ spermatogonia in cultures. To promote differentiation, cultures were shifted to the same medium containing all-trans-retinoic acid (ATRA), which formulated SD^FA^ Medium (Table 1). Spermatogonia were allowed to differentiate in SD^FA^ Medium for 6 additional days prior to analysis. Three ERBB2 inhibitor types were tested with and without ATRA: (1) the small molecule Lapatinib, (2) an ERBB2-neutralizing antibody, and (3) soluble extracellular ERBB3/ERBB4 domains (to deplete NRG-family ligands). Each inhibitor modality effectively and selectively disrupted clonal development of differentiating spermatogonial syncytia (SALL4^−^, DAZL^+^) on MEFs in SD^FA^ Medium (Figures 3a and b; Supplementary Figure S2).

Lapatinib treatment did not affect relative numbers of A_s_, A_pr_ and A_al_ spermatogonia that incorporated 5-ethynyl-2’-dexoyuridine (EDU) (7.5-h pulse) into newly synthesized DNA after 4 days in SD^FA^ Medium on MEFs (Figure 3c). It is important to note that a shorter preincubation time in SG^F^ Medium and a shorter incubation time in SD^FA^ Medium was applied to better capture viable spermatogonial syncytia incorporating EDU in the presence of Lapatinib (Figure 3c). Lapatinib also did not influence relative numbers of functional spermatogonial stem cells maintained in MEFs under basal conditions without ATRA (Figures 3d and e). Lapatinib did reduce a GDNF-dependent increase in stem cell numbers (~twofold) back to basal levels in the absence of ATRA (Figure 3e). Differentiating spermatogenic cells isolated following treatment with ATRA, either with or without Lapatinib, were effectively depleted of spermatogonial stem cells (Figure 3e). Because Lapatinib did not change rates of spermatogonial development into syncytia based on EDU incorporation (Figure 3c) and did not change basal spermatogonial stem cell numbers/culture (Figure 3e), an ERBB2-dependent mechanism was identified that selectively regulated survival of differentiating spermatogonia.

### ERBB2 signals soma-free survival of differentiating spermatogonia

**Figure 4:**
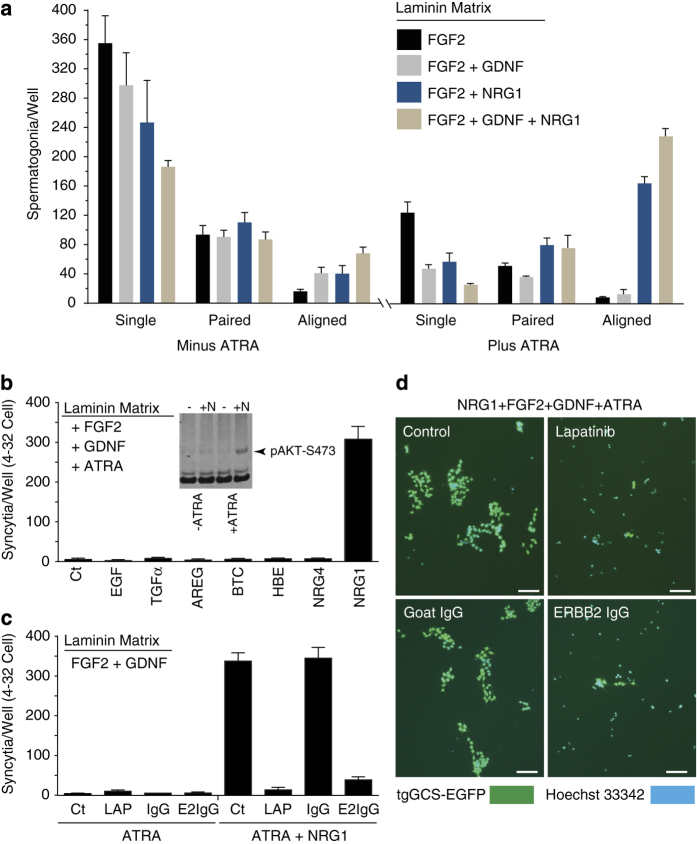
NRG1/ERBB3/ERBB2-dependent growth of differentiating spermatogonia. (**a**) ATRA and Neuregulin-1 stimulate development of aligned spermatogonia. tg*GCS-EGFP*^+^ spermatogonial scored/well after 1 week of culture in SG^F^ Medium (Table 1) on laminin and then 6 additional days with and without ATRA (3 *μ*M) in combination with GDNF (0.1 nM) and/or NRG1 (5 nM). Representative of duplicate *in vitro* spermatogenesis colony-forming assays scored after 2 days in SG^F^ Medium and 6 days in SD Medium (±S.E.M., triplicate wells). (**b**) Effects of various ERBB-family receptor agonists on soma-free rat spermatogonial cultures on laminin. Respective ERBB agonists were substituted for NRG1 in SD Medium. Representative of duplicate *in vitro* spermatogenesis colony forming assays (±S.E.M., triplicate wells). Inset: western blotting for pAKT-S473 (arrowhead) and p42/44 (lower bands) in spermatogonia in SG^F^ Medium for 2 days, prior to culturing with and without ATRA for 4 days, and then treating with and without NRG1 for 20 min before lysing. (**c**) ERBB2 inhibitors disrupt NRG1-dependent spermatogonial development on laminin. Representative of duplicate colony-forming assays scored (±S.E.M., triplicate wells). (**d**) Images of NRG1-dependent syncytial growth of tgGCS-EGFP^+^ spermatogenic cells on laminin. Scale, 100 *μ*m. Nuclei labeled with Hoechst 33342 dye.

Next, we tested spermatogonial growth in SG^F^ Medium supplemented with distinct combinations of GDNF, NRG1 and ATRA on a laminin matrix (Figure 4a). Under each condition tested, ATRA did not sustain development of spermatogonial syncytia without NRG1 (Figure 4a). However, with NRG1, ATRA effectively promoted syncytial growth (Figure 4a). The combination of FGF2, GDNF, NRG1 and ATRA (SD Medium; Table 1) was most effective at supporting accumulation of spermatogonial syncytia (A_al_), as well as increasing their relative numbers over that of A_s_ spermatogonia (Figure 4a). Spermatogonial development in SD Medium on laminin appeared similar to SG^F^ Medium supplemented with ATRA and MEFs (Figures 3a and b).

To test additional ERBB-family receptors for spermatogenic effects, we compared the ability of highly selective ERBB1, ERBB3 and/or ERBB4 agonists to support soma-independent spermatogonial differentiation on laminin in SD Medium. Consistent with effects of knocking out *Erbb3* in the germline^32^ and detection of ERBB2 and ERBB3 in spermatogonia (Figure 1b), only the ERBB3 agonist NRG1 stimulated clonal development of differentiating spermatogenic cells in SD Medium on laminin (Figure 4b). Several other polypeptide agonists that selectively activate ERBB1 and/or ERBB4 were ineffective compared with NRG1 (Figure 4b).

Accordingly, ATRA upregulated the ability of NRG1 to stimulate phosphorylation of the survival factor AKT1 (Serine 473) in differentiating spermatogonia (Figure 4b; inset). And, consistent with spermatogonial cultures on MEFs, NRG1-dependent development of differentiating spermatogonia on laminin was effectively blocked by Lapatinib or the ERBB2-neutralizing antibody (Figure 4c). Because the tg*GCS-EGFP* transgene is a viable germ cell marker,^39^ it became clear by co-labeling nuclei with Hoechst 33342 dye in the absence of feeder cells that NRG1 was required for survival of differentiating spermatogonia on laminin (Figure 4d). Thus ERBB agonist selectivity (Figure 4b), ERBB2 inhibitor sensitivity (Figure 4c) and NRG1-dependent AKT-Ser473 phosphorylation (Figure 4b) delineated a NRG1/ERBB3/ERBB2 signaling pathway in SD Medium that supported soma-independent survival of differentiating spermatogonia.

### ERBB2/3 and KIT regulate alternate spermatogenic pathways *in vitro*

Despite an inability to support spermatogonial differentiation in culture, *Erbb3*-null germlines robustly supported spermatogenesis in recipient testes,^32^ which indicated that spermatogonial cultures lacked spermatogenesis growth factors present in testes. Thus we sought an alternate pathway that could rescue spermatogenic development on the *Erbb3*-null background in SD Medium.

Because KIT is expressed on differentiating spermatogonia,^24,25,40^ and KIT was required for spermatogonial differentiation *in vivo*,^22,41,42^ KITL provided a top candidate growth factor to rescue *Erbb3*-null germline defects. Moreover, KITL elicited antiapoptotic effects on differentiating spermatogonia, spermatocytes and spermatids in rat seminiferous tubule cultures.^43^ Sterilizing *Kit* mutations have long been linked to the *dominant white-spotting* (*W*) locus in mammals.^23^ Yet, by unknown mechanisms, mouse spermatogonial lines harboring sterilizing W^v^/W^v^ *Kit* mutations supported spermatogenesis in recipient testes on a W/W^v^ C57BL/6 genetic background.^44^

**Figure 5:**
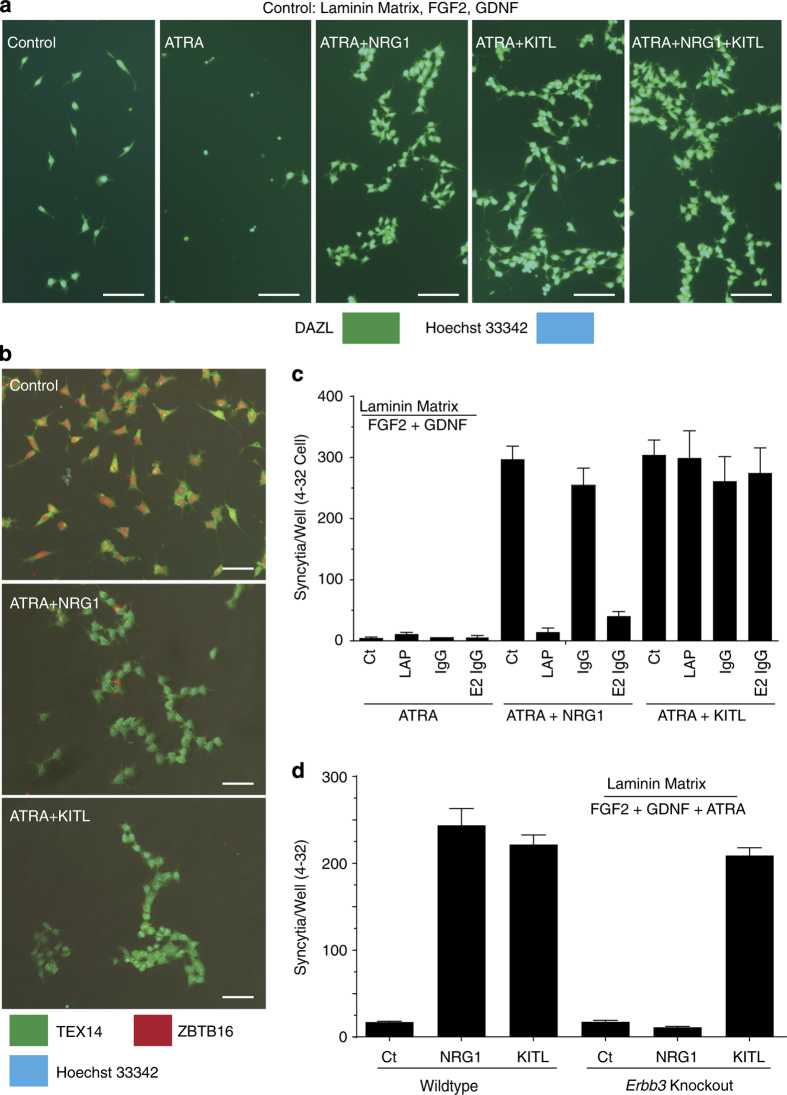
KITL supports spermatogonial differentiation independent of ERBB2. (**a**) Images of NRG1- and KITL-dependent spermatogonial differentiation after 2 days in SG^F^ Medium and 6 days in SD Medium. Spermatogonia are labeled with the germ cell marker DAZL (green); nuclei labeled with Hoechst 33342 dye. Scale, 100 *μ*m. (**b**) Spermatogonia cultured as in panel (**a**) labeled with antibodies to the germ cell marker TEX14 (green) and a marker for undifferentiated type A spermatogonia, ZBTB16 (red); nuclei labeled with Hoechst 33342 dye. Scale, 50 *μ*m. (**c**) ERBB2 inhibitors selectively disrupt NRG1-dependent but not KITL-dependent spermatogonial development on laminin. Representative of duplicate colony-forming assays (±S.E.M., triplicate wells). (**d**) KITL rescues spermatogonial syncytial growth of *Erbb3*-deficient germlines on laminin. Representative of duplicate colony-forming assays (±S.E.M., triplicate wells).

**Figure 6:**
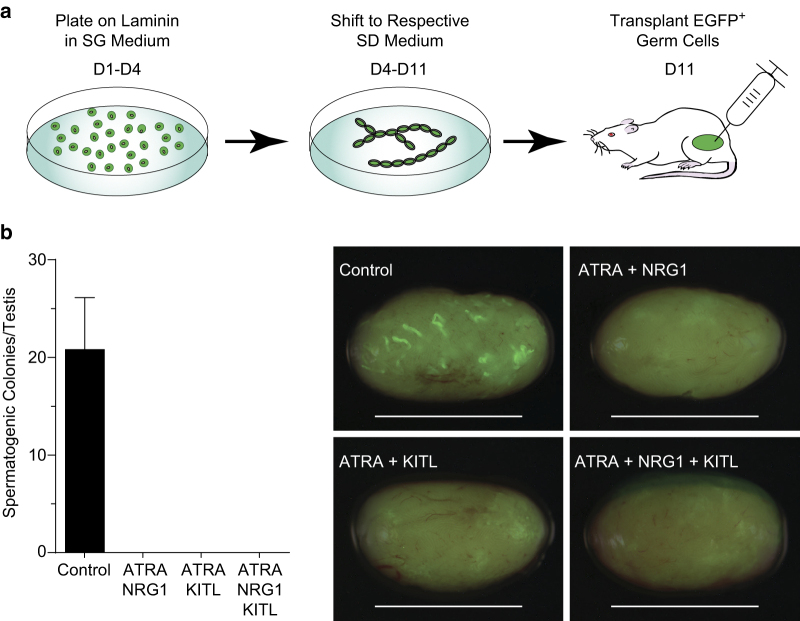
Spermatogonia cultured in SD Medium lose germline stem cell activity. (**a**) Diagram of spermatogonial culture experiment on laminin matrix prior to transplanting respective spermatogonial cultures into recipient rat testes (*n*=5 rats/condition with right testis transplanted; *P*<0.0001 control *versus* each test condition, multiple *t*-tests). (**b**) Left: Relative numbers of spermatogenic colonies generated/testis/1000 donor germ cells (tg*GCS-EGFP*^+^) harvested from cultures described in panel (**a**) after 1 week in respective SD Media containing NRG1 and/or KITL (D4–D11). Donor ‘Control’ cultures were maintained on laminin in SG Medium the full 10 days (D1–D11) prior to transplantation. Recipients analyzed 28 days posttransplantation. Right: Images of recipient rat testes after being transplanted with spermatogonia from each respective culture condition. Scale, 1 cm.

Indeed, here KITL effectively supported retinoic acid-induced spermatogonial differentiation (DAZL^+^, TEX14^+^, PLZF^−^) on laminin (Figure 5a). The combination of NRG1 and KITL promoted formation of large spermatogenic syncytia containing >32 cells after the 6-day culture period (Supplementary Figure S3). KITL-dependent spermatogonial development was not blocked by ERBB2 inhibitors (Figure 5b), and KITL fully rescued spermatogonial differentiation on the *Erbb3*-null background (Figure 5c). Moreover, NRG1- and/or KITL-dependent spermatogonial growth in SD Medium effectively depleted spermatogonial stem cells from cultures (Figure 6). Thus NRG1 and KITL were each essential for soma-free, clonal development of differentiating spermatogenic cells in SD Medium.

## Discussion

Lack of success at culturing mammalian stem cells through spermatogenesis in defined systems has fueled hypotheses that the complexity of specific germ cell and somatic cell relationships in testes is essential for the process spermatogenesis, which explains why highly pure mammalian spermatogonia have not developed into meiosis, much less through meiosis without somatic cells. Here, we demonstrate that despite intricate germ cell and somatic cell associations in the seminiferous epithelium,^1^ it remains possible to molecularly dissect actions of spermatogenic growth factors in the ‘test tube’ following differentiation of primary spermatogonial stem cell lines. Systematic addition of candidate factors to differentiating spermatogonial clones in culture without somatic cells provides a strategy to derive haploid gametes *in vitro*.

**Figure 7:**
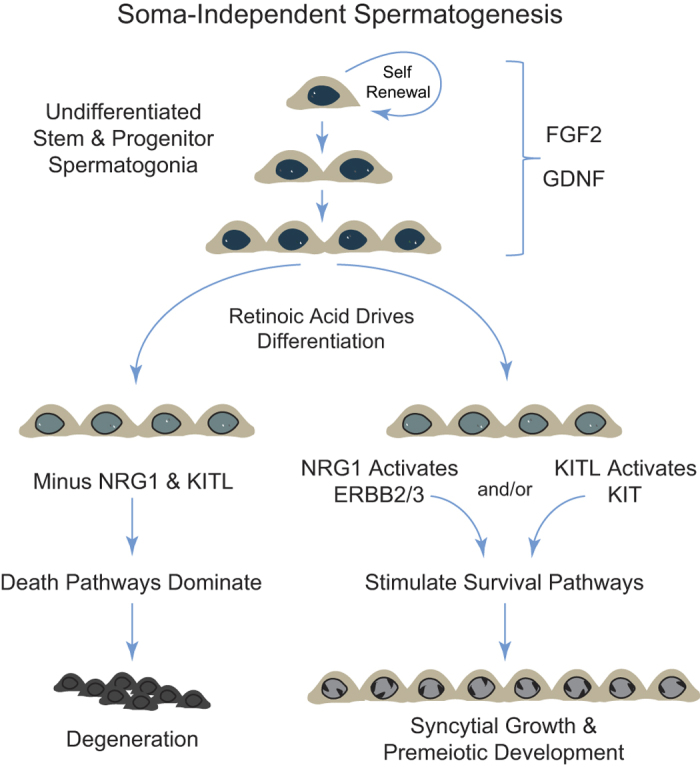
Soma-independent development of differentiating spermatogonia. FGF2 and GDNF support spermatogonial stem cell renewal and syncytial growth of early undifferentiated spermatogonial progenitors (DAZL^+^, TEX14^+^, ZBTB16^+^) in culture on laminin. Retinoic acid acts in the germline to drive transformation of undifferentiated spermatogonia into nascent differentiating spermatogonia. Polypeptide growth factors NRG1 and KITL are required for survival of differentiating spermatogonia on laminin without somatic cells. In the absence of NRG1 and KITL, ATRA drives undifferentiated spermatogonia to undergo development into syncytia of ~4-16 DAZL^+^, TEX14^+^, ZBTB16^−^ spermatogenic cells that degenerate.

By combining pharmacological and genetic approaches to study a defined spermatogonial culture system, we delineated a NRG1/ERBB3/ERBB2 signaling pathway in the germline that supported premeiotic steps of spermatogenesis *in vitro* (Figure 4). Once ERBB-family subunits that transduced NRG1 signaling in spermatogonia were elucidated in a defined system, we were able to test our hypothesis that an alternate, ERBB-independent pathway could support spermatogonial development *in vitro* without somatic cells. Based on studies where testicular KIT activity was required for spermatogonial development,^22,23,42^ we demonstrated that a primary candidate, KITL, could in fact stimulate soma-free syncytial growth of differentiating spermatogonia, which phenocopied NRG1-dependent effects on germ cell development *in vitro* (Figure 7).

In mouse testes, ATRA rapidly signaled KIT synthesis by directing repressed *Kit* mRNAs into polysomes.^45^ In mouse undifferentiated spermatogonia cultured on fibroblasts, ATRA upregulated *Kit* and promoted *Kit* translation by repressing germline miRNAs 221/222.^46^ Moreover, in a separate study, miRNA 146 was found to block accumulation of *Kit* transcripts/KIT protein in undifferentiated spermatogonia on fibroblasts, and ATRA aptly downregulated miRNA 146.^47^ Here we found that most NRG1-like activity supporting ATRA-induced spermatogonial differentiation on fibroblasts was ERBB2 dependent (Figures 3a and b). Thus ERBB2 was either acting downstream of KITL or via an independent pathway in spermatogonia, with the later scenario being the case (Figure 5).

Control to dissect cellular processes using a defined culture system was exemplified by NRG1’s mechanism of action on spermatogonial development reported here, as compared with NRG1’s effects on spermatogonial development using a complex culture system.^48^ NRG1 was purified from MEF conditioned medium by its ability to synergistically stimulate syncytial growth of differentiating spermatogonia with GDNF and serum.^48^ In serum-containing medium, NRG1’s effectiveness on spermatogonial syncytia growth was fully dependent on GDNF,^48^ whereas here NRG1 effectively stimulated spermatogonial syncytial growth independent of GDNF but in combination with ATRA and FGF2 (Figure 4a). Thus it should be determined whether serum factors biologically distinct from ATRA can drive spermatogonial differentiation in culture with NRG1 and GDNF.^48^

Robust spermatogonial differentiation from stem spermatogonia under highly controlled culture conditions provides a platform to identify factors that can stimulate germ cells to undergo meiosis *in vitro*. Our studies highlight how alternate signaling pathways regulated by NRG1 and KITL can support spermatogonial development *in vitro* (Figure 7), which conceivably contribute to the genetic robustness of spermatogenesis *in vivo*.^32,44^ Existence of alternate growth factor pathways that act directly in spermatocytes will prospectively facilitate discovery of additional factors that cooperate with NRGs, KITL and/or retinoic acid to promote meiotic progression from differentiating spermatogonia in the culture dish.

## Materials and Methods

### Animal care and use

Protocols for the use of rats in this study were approved by the Institutional Animal Care and Use Committee at UT-Southwestern Medical Center in Dallas, TX, USA as certified by the Association for Assessment and Accreditation of Laboratory Animal Care International. Rats used for this study were housed in individually ventilated, Lab Products 2100 cages (Seaford, DE, USA) in a dedicated room with atmosphere controls set to 72 °F, 45–50% humidity during a 12-h light/dark cycle (i.e., light cycle=0600–1800 hours, Central Standard Time adjusted for daylight savings time). Rats were fed Teklad LM-485 mouse/rat diet, 5% fat diet (cat no. 7912, Harlan, Inc., Indianapolis, IN, USA) and a continuous supply of reverse osmosis water. Wild-type Sprague Dawley rats were from Harlan, Inc. (Houston, TX, USA) and Brown Norway rats were form Charles Rivers, Inc. (Kingston, NY, USA).

### Spermatogonial culture

Rat spermatogonial lines were derived from freshly isolated laminin-binding (LB) spermatogonia and maintained using spermatogonial culture medium (SG Medium), as previously described.^49,50^ The base SG Medium formulation contained: Dulbecco’s Modified Eagle’s Medium:Ham’s F12 Nutrient Mixture (1 : 1), 6 ng/ml GDNF, 6 ng/ml FGF2, 100 *μ*M 2-mercaptoethanol, 6 mM L-glutamine, and a 1x concentration of the B27-Minus Vitamin-A Supplement solution. Spermatogonia were sub-cultured on feeder layers of MEFs.^49,50^ Prior to *in vitro* or *in vivo* spermatogenesis colony-forming assays, spermatogonia were harvested from MEFs and incubated for 3–4 h on gelatin-coated plates in SG Medium to remove feeder cells.

### Isolating undifferentiated type A spermatogonia

Seminiferous tubules were isolated from testes of 23–24-day-old homozygous *SD*-*Tg*(*ROSA-EGFP*)*2-4*^*Reh*^ or WT Brown Norway (Charles Rivers, Inc.) rats. The most advanced germ cell types in 23-day-old male rats are spermatogonia and spermatocytes.^51^ Rats of the *SD*-*Tg*(*ROSA-EGFP*)*2-4^Reh^* line were produced by pronuclear injection and are referred to as *GCS-EGFP* rats, because they exhibit germ cell-specific expression of enhanced green fluorescent protein (EGFP).^39^ Tubules were enzymatically and mechanically dissociated into a cellular suspension to generate cultures of testis cells in serum-containing medium.^52,53^ The testis cell cultures were then used to isolate enriched populations of LB spermatogonia by established methods^52,53^ that first remove >95% of somatic testis cells from the germ cell population by negative selection on plastic and collagen, before positive selection for the spermatogonial stem cells based on their ability to bind to laminin.^53^ By this procedure, freshly isolated LB germ cells contain >90% undifferentiated, type A spermatogonia (PLZF^+^, DAZL^+^) in the single (~88%) or paired (~12%) cell state.^48,54^ Note, fractions of LB spermatogonia isolated by this procedure contain ~4% somatic cells and ~5% differentiating spermatogonia/early spermatocytes; and fractions of laminin-non-binding spermatocytes were >95% differentiating spermatogonia/early spermatocytes.^54^

### *In vitro* spermatogenesis colony-forming assay

Spermatogenic colonies formed *in vitro* were scored after plating ~1.5×10^3^ spermatogonia/well (0.96 cm^2^) in spermatogonial differentiation medium (SD Medium) at 36.5 °C, 5% CO_2_. Wells were precoated with ~5 *μ*g/cm^2^ laminin as described.^49^ SD Medium is a modified formulation of the serum-free, SG Medium.^50^ To make SD Medium, SG Medium was modified to contain 6 ng/ml FGF2 (PGF0023, Life Technologies, Inc., Grand Island, NY, USA), 2 ng/ml GDNF (512-GF, R&D Systems, Inc., Minneapolis, MN, USA), 40 ng/ml NRG1*β*1 T176-K246 (396-HB, R&D Systems, Inc.) and 3 *μ*M ATRA (R2625, Sigma, Inc., St. Louis, MO, USA). KITL (455-MC-010, R&D Systems, Inc.) was supplemented with SD Medium at 100 ng/ml with or without NRG1 in respective experiments.

Under standard, soma-free bioassay conditions using laminin matrix, on ‘day 1’ (d1) spermatogonia were seeded in SG^F^ Medium (without GDNF; Supplementary Table S1) and, except where noted, were maintained for 48 h (*n*=3 wells/line) before shifting cultures into SD Medium on d3. Medium was changed again on d5 and d7 using fresh SD Medium. On d9 (i.e., 6 days in SD Medium), medium was removed and cells were fixed in 4% paraformaldehyde, sodium phosphate buffer (0.1 M, pH 7.2) for 20–30 min on ice. Spermatogenic units were scored/well (*n*=3 wells/line/experiment, ±S.E.M.) by counting numbers of ‘Single’, ‘Paired’ and ‘Aligned’ spermatogenic cells. One ‘Aligned’ Spermatogenic Unit=1 syncytium of tgGCS-EGFP^+^ spermatogenic cells containing 4–32 Hoechst 33342-labeled nuclei. The terms ‘Aligned’ or ‘A_al_’ were based purely on morphology and thus inclusive of ‘undifferentiated’ (ZBTB14^+^ or SALL4^+^) and ‘differentiating’ (ZBTB14^−^ or SALL4^−^) spermatogonia in graphs.

Cells were postfixed in methanol for 5 min on ice and then washed 2× with phosphate-buffered saline (PBS) prior to fluorescence labeling with Hoechst 33342 dye and/or antibodies to ZBTB14 (0.2 *μ*g/ml, Clone 2A9, 39987, Active Motif, Inc., Carlsbad, CA, USA), SALL4 (0.2 *μ*g/ml, H0005716-M03, clone 6E3; Abnova, Taipei City, Taiwan), DAZL ~5 nM^53^ and/or TEX14 ~10 nM.^54^ Spermatogenic colony scoring and imaging was conducted using an IX70, Olympus fluorescence microscope (Olympus Inc., Waltham, MA, USA) equipped with the Simple-PCI software (C-Imaging Systems, Compix, Cranberry Township, PA, USA). As quality control, representative tgGCS-EGFP^+^ spermatogonial lines were verified as being germ cells by independently labeling with DAZL IgG and TEX14 IgG prior to (d3) and following differentiation in SD Medium (d9). For experiments utilizing MEFs in Figure 3 and Supplementary Figure S2, spermatogonia were preincubated in SG^F^ Medium on MEFs for 6–8 days, as indicated in legends. Longer preincubation times in SG^F^ Medium increased the percentage of A_s_ spermatogonia in cultures. Lapatinib (L-4804) was from LC Laboratories (Woburn, MA, USA); ERBB2-neutralizing IgG (AF1129), ERBB3 (348-RB) and ERBB4 (1131-ER) Fc chimeric proteins were from R&D Systems.

### *In vivo* spermatogenesis colony-forming assay

WT Sprague Dawley rats at 12 days of age were injected (i.p.) with 12 mg/kg busulfan (4 mg/ml in 50% DMSO) and then used as recipient males at 24 days of age. Busulfan is a spermatogonial toxin commonly used to kill spermatogonia in recipient rat testes prior to transplantation, because it increases the colonization efficiency by the donor stem cells.^53,55,56^ Donor cells were loaded into injection needles fashioned from 100 *μ*l glass capillary tubes at 10^3^ cells/65 *μ*l SG Medium containing 0.032% (wt/vol) trypan blue, and then the entire volume was transplanted into the seminiferous tubules of anesthetized rats by retrograde injection through the rete testes.^56,57^ Recipient males were analyzed 28 days posttransplantation, and colonies of spermatogenesis expressing the tg*GCS-EGFP* transgene were scored using an IX70, Olympus fluorescence microscope (Olympus Inc.) equipped with the Simple-PCI software (C-Imaging Systems, Compix).^54^ Images of transgene expression in whole testes were captured using a Nikon SMZ1500 fluorescence stereomicroscope equipped with the ACT-1 imaging software (Nikon Instruments, Inc., Melville, NY, USA).^54^

### Immunofluorescence labeling in testis sections

Testes were isolated from wild-type Sprague Dawley rats (Harlan, Inc.) and fixed for ~18 h at 4 °C in 0.1M sodium phosphate buffer, pH 7.2, containing 4% paraformaldehyde. Fixed testes were equilibrated through a 10, 18 and 25% sucrose (wt/v, dissolved in 1× PBS; Life Technologies, Inc., cat. no. 14040-182) gradient by sequential overnight incubations (~24 h) at 4 °C in 15 ml of each respective sucrose solution. Once equilibrated to 25% sucrose, testes were embedded in tissue-freezing medium (Electron Microscopy Sciences Inc., Hatfield, PA, USA, cat. no. 72592) and frozen using a Shandon Lips haw cryo-bath (Pittsburgh, PA, USA, cat. no. 45972). Frozen testes were used to prepare a parallel series of 8-*μ*m cryo-sections. Frozen sections were stored at −40 °C until use in antibody-labeling assays. Prior to antibody labeling, sections were equilibrated in air to ~22–24 °C for 15 min, hydrated in PBS (Sigma, cat. no. D8537) at 22–24 °C for 10 min, heat treated at 80 °C for 8 min in 10 mM sodium citrate (pH 6.0) and then incubated for 1 h at 22–24 °C in blocking buffer (Roche Blocking Reagent (1% v/v) diluted in 0.1M sodium phosphate buffer, pH 7.2, containing Triton X100 (0.1% v/v)). Sections were then treated for 18–24 h at 22–24 °C with respective antibodies diluted in blocking buffer at the following concentrations: Anti-ERBB2, 0.25 *μ*g/ml (Santa Cruz Inc., Dallas, TX, USA, Neu C18, sc-284); Anti-ERBB2, used at 1/150 dilution (Cell Signaling Technology Inc., Danvers, MA, USA, 29D8, 2165); Anti-PLZF, 0.2 *μ*g/ml (EDM Millipore, Billerica, MA, USA, cat. no. OP128, clone ID 2A9); anti-SALL4, 0.2 *μ*g/ml (Abnova cat. no. H0005716-M03, Clone ID 6E3); NRG1a1 (R&D Systems, Inc., cat. no. AF296NA); and NRG1b1 (R&D Systems, Inc., cat. no. AF396NA). After treatment with primary antibodies, sections were washed three times for 10 min/wash in 50 ml PBS and then incubated for 40 min at 22–24 °C with respective AlexaFluor594 (Life Technologies, Inc.), or AlexaFluor488 (Life Technologies, Inc.) secondary antibodies diluted to 4 *μ*g/ml in PBS containing 5 *μ*g/ml Hoechst 33342 dye (Molecular Probes, cat. no. H3570). After treatment with secondary antibodies, sections were washed three times at 10 min/wash in 50 ml PBS. After the third wash in PBS, sections were cover-slipped for viewing using Fluorogel mounting medium (Electron Microscopy Sciences, cat. no. 17985-10). Images were acquired using an IX70 Olympus fluorescence microscope (Olympus Inc.), as described above in 'Immunofluorescence labeling on seminiferous tubules' section. Labeled sections were cover-slipped using Fluorogel mounting medium and images were acquired as described above.

### Immunofluorescence labeling in seminiferous tubule whole mounts

Seminiferous tubules were dissected from rat testes in 10-cm dishes containing Dulbecco modified Eagle’s medium: Ham’s F12 medium 1 : 1 (DHF12 Medium; Sigma, cat. no. D8437) at ~22–24 °C. Individual tubules were teased apart from testes by blunt dissection, rinsed in fresh DHF12 medium and fixed for 1 h at 4 °C in 20 ml 0.1M Sodium phosphate buffer, pH7.2, containing 4% paraformaldehyde. Optimal co-labeling with antibodies was obtained by suspending the paraformaldehyde-fixed tubules in ice-cold methanol and then immediately incubating them for additional 20 min at −20 °C (applied to obtain spermatogonial frequency data from 104–126d rats). Following fixation, tubules were washed three times in 20 ml PBS for ~10 min/wash at 22–24 °C and then stored at 4 °C for up to 1 week prior use. For indirect labeling with antibodies, 0.5–2.5 cm long tubule pieces were aliquoted into microfuge tubes containing 800 *μ*l and 10 mM sodium citrate (pH 6.0) and incubated at 80 °C for 6–8 min. After heat treatment, tubules were immediately washed by dipping in PBS at 22–24 °C and then incubated for 1 h at 22–24 °C in blocking buffer before treating with respective primary and secondary antibodies as described above in 'Immunofluorescence labeling in testis' section.

### EDU incorporation

Spermatogonia were passaged into SG^F^ Medium and maintained for 48 h prior to treating cells with SG^F^ Medium containing 3 *μ*M ATRA for 4 days. EDU was added to culture medium at a final concentration of 10 *μ*M and incubated for 7.5 h. Cells were fixed in 4% paraformaldehyde followed by methanol as described previously for immunocytochemistry. EDU was detected using the Click-it Edu Alexa-Fluor 594 Imaging Kit (Life Technologies Inc., cat. no. C10339).

### Western blotting

Whole-cell lysates were prepared in lysis buffer (50 mM Tris, 150 mM NaCl, 1 mM EDTA, 1% IGEPAL CA-630) with protease inhibitors (Complete EDTA-free protease inhibitor tablets, Roche Applied Science, Inc., Indianapolis, IN, USA, cat. no. 11836170001) and phosphatase inhibitors (PhosSTOP, Roche Applied Science, Inc., cat. no. 4906845001). Samples were clarified by centrifugation in a microfuge at 4 degrees for 10 min. Samples were run on SDS-PAGE gels under reducing conditions and transferred to nitrocellulose. Blots were probed for ERBB1 (Santa Cruz, Inc., cat. no. sc-03), ERBB2 (Lab Vision, Inc., cat. no. MS-599-P1), ERBB3 (Santa Cruz, Inc., cat. no. sc-285), ERBB4 (Cell Signaling Technology, Inc., cat. no. 4795), RET (R&D Systems, Inc., cat. no. AF482), NRG1*α*1 (R&D Systems, Inc., cat. no. AF296NA), NRG1*β*1 (R&D Systems, Inc., cat. no. AF396NA), phospho-AKT-S473 (Cell Signaling Technology, Inc., cat. no. 9271), phospho-P42/44 MAPK (Cell Signaling Technology, Inc., cat. no. 9101) and alpha-tubulin (Biogenex, Inc., Fremont, CA, USA, cat. no. MU121-UC). Secondary antibodies labeled with IRDye 800CW or 680LT were from Li-Cor Biosciences, Inc. (Lincoln, NB, USA). Blots were imaged on Odyssey Classic Quantitative Fluorescence Imaging System, Model 9120, also from Li-Cor Biosciences.

### RT-PCR and qtPCR analysis of testis cell transcripts

Total RNA was isolated from testis cell sampless using the RNAqueous Micro Kit (Life Technologies, Inc.). RNA concentration in each isolated sample was measured using the RiboGreen Kit (Life Technologies, Inc.). Fifty nanograms of RNA from each cell sample was primed in a 20 *μ*l reaction mix containing Oligo(dT) primers and Superscript III (Life Technologies, Inc.), according to the manufacturer’s protocol. Gene expression was analyzed by PCR using PfuTurbo in UltraHF Buffer from Stratagene, Inc. (Santa Clara, CA, USA), qtPCR conditions were as described,^50^ and RT-PCR conditions were as follows: 1 cycle, 2 min at 95°; 4 cycles 30 s at 95°, 45 s at 65°, 1 min at 68°; 32 cycles, 30 s at 95°, 45 s at 60°, and 1 min at 68°. PCR primers were designed to rat reference sequences in NCBI using Primer/Blast:[Table tbl2]

### DR4 fibroblast feeder layer preparation

Primary stocks of DR4 MEFs were from ATCC, Inc. (Manassas, VA, USA), and expanded after plating into Dulbecco’s modified Eagle’s medium supplemented with 1.5 g/l sodium bicarbonate and 15% heat-inactivated FBS (MEF medium) at 37 °C/5% CO_2_ for up to four passages following their thawing and initial plating from the vial received from the manufacturer. Following expansion, secondary stocks of MEFs are irradiated (100 Gy) and then cryo-preserved in liquid nitrogen for future use in Recovery Cell Culture Freezing Medium (Life Technologies, Inc.) according to the manufacturer’s protocol. Tissue culture dishes were precoated with a solution of sterile 0.1% gelatin for 1 h at room temperature and rinsed once with sterile PBS before plating MEFs. Prior to use for culture with spermatogonia, irradiated MEFs were plated into gelatin-coated dishes (~6.8×10^4^ cells/cm^2^) in MEF medium for 16–48 h, rinsed 1× with PBS and then preincubated in SG Medium for an additional 16–48 h. The SG Medium used for preincubation is then discarded and spermatogonia are passaged onto the MEFs in fresh SG Medium.

## Figures and Tables

**Table 1:** Spermatogonial culture and differentiation media

**Spermatogonial medium** a	**Growth factor supplement**
SG Medium^b^	GDNF FGF2
SG^F^ Medium^b^	FGF2
SD Medium^c^	GDNF FGF2, ATRA NRG1 and/or KITL
SD^FA^ Medium^c^	FGF2, ATRA

a Base medium=DMEM:HAMS-F12 (1 : 1) nutrient mixture, 6 mM L-glutamine, 100 *μ*M 2-mercaptoethanol, 1× B27 minus vitamin A supplement, 1× antibiotic/antimycotic solution; Wu *et al*.^50^

b SG Medium=spermatogonial culture medium: 6 ng/ml GDNF and 6 ng/ml FGF2.

c SD Medium=spermatogonial differentiation medium: 2 ng/ml GDNF, 6 ng/ml FGF2, 3 *μ*M ATRA, 40 ng/ml NRG1*β*1 (T176-K246), and/or 100 ng/ml KITL.

**Table 2 tbl2:** 

	Forward primers	Reverse primers
Nrg1	5′-TGCCTCCCAGATTGAAAGAAATG-3′	5′-GTTAATGTTCTCATGCGACAG-3′
Type I: NRG1	5′-ATCTTCGGCGAGATGTCTGA-3′	
Type II:GGF2	5′-AACCTCAAGAAGGAGGTCAG-3′	
TypeIII:N-term dom	5′-TGAAGTGGGTATTTGTGGAC*-*3′	
Nrg1-alpha-1 form		5′- TTGGGTTTGGACTTTCATGGGTAC-3′
Nrg1-beta-1 form		5′- TAGAAGCTGGCCATTACGTAG-3′
Nrg1-sec form		5′- GTTAATGTTCTCATGCGACAG-3′
Nrg1-transmem		5′- GTTTTACAGGTGAATCTATGTG-3′
Nrg2	5′-AGATGAAGAGTCAGACAGGAG-3′	5′-ATGTTCTCAGCCTCACAGACG-3
Nrg3	5′-AACAGATCCGGATTCTGACTG-3′	5′-TGCACAGATCCCTACATCTCC-3′
Nrg4	5′-ACATGACTCCCATGCATGAC-3′	5′-TACTCAGGATTCTCTGCCAC-3′
Cspg5	5′-TTTCTGCAGGTGTAACACCC-3′	5′-TTGGGTGACATGGAGTTCTGG-3′
Egf	5′-AAGCAGCTATCAGAGAGCTC-3′	5′-TGATGGTGGAATCCAGCAGC-3′
Tgfa	5′-AAGTGTAGCCTGCTGCTCAG-3′	5′-AGACACTAAAGCTGAGGTCC-3′
Hbegf	5′-AGTGCAGATACCTGAAGGAG-3′	5′-AGAAGTCTTCATGGCTGCTGG-3′
Areg	5′-TGTGGACGACTCAGTCAGAG-3′	5′-TGGATAGGTCGCTGTCATCC-3′
Btc	5′- ATCGAAAACCCACTTCTCTCGG-3′	5′-AAGAGGATGACAGCAGGTGCAG-3′
ErbB1	5′-TCTTGAGCTCTCTGAGTGC-3′	5′-AAGAAGTCCTGCTGGTAGTC-3′
ErbB2	5′-TGTTTGATGGTGACCTGGC-3′	5′-TGCTCCGATGAGTTCTGG-3′
ErbB3	5-AACTAGCCAATGAGTTTACC-3′	5′-ACAGAACTGAGACCTACCG-3′
ErbB4	5′-ACATGACTCCCATGCATGAC-3′	5′-TACTCAGGATTCTCTGCCAC-3′
Dazl	5′-ACTTATCATGTGCAGCCACG-3′	5′-AGACAAGAGACCACTGTCTG-3′
Kncl	5′-CTCGCTGTGGCAGGAAACA-3′	5′-VAAGAGGGCCTTGCCTTTCTC-3′
Inl3	5′-CTAGGGATCCTCCAAGGCAAT-3′	5′-CACCCAGCAAGACCTTTTGG-3′
Gapdh	5′-ATGATTCTACCCACGGCAAG-3′	5′-GCTAAGCAGTTGGTGGTGCA-3′

## Abbreviations

A_s_: A-single
A_pr_: A-paired
A_al_: A-aligned
AKT: v-akt murine thymoma viral oncogene homolog 1
AREG: amphiregulin
ATRA: all-trans-retinoic acid
BTC: betacellulin
CSPG5: chondroitin sulfate proteoglycan 5
DAZL: Deleted in azoospermia
EDTA: ethylenediaminetetraacetic acid
EDU: 5-ethynyl-2′-deoxyuridine
EGF: epidermal growth factor
ERBB: erythoblastoma virus B homolog
FGF2: fibroblast growth factor 2
tgGCS-EGFP: germ cell specific-enhanced green fluorescence protein-encoding transgene
GDNF: glial cell line derived neurotrophic factor
GFRa1: GDNF family receptor alpha 1
HBE or HBEGF: heparin-binding-epidermal growth factor
IGEPAL: octylphenoxypolyethoxyethanol
KIT: v-kit Hardy–Zuckerman 4 feline sarcoma viral oncogene homolog
KITL: KIT ligand
MEF: mouse embryonic fibroblast
miRNA: microRNA
NRG: neuregulin
PBS: phosphate-buffered saline
qtPCR: semi-quantitative polymerase chain reaction
RT-PCR: reverse transcription-polymerase chain reaction
RET: ret proto-oncogene
SALL4: spalt-like transcription factor 4
SD medium: spermatogonial differentiation medium
SG medium: spermatogonial culture medium
TEX14: testis expressed gene 14
TGFα: transforming growth factor alpha
WNT: wingless-type MMTV integration site family
ZBTB16: zinc finger and BTB domain containing 16.
