# Supplementary material for: NRG1 and KITL signal downstream of retinoic acid in the germline to support soma-free syncytial growth of differentiating spermatogonia
Source: Cell Death Discov. 2015 Oct 5;1:15018–. doi: 10.1038/cddiscovery.2015.18 (PMC4613782; doi:10.1038/cddiscovery.2015.18)
Supplement: Supplementary Figures [file cddiscovery201518-s1.pdf]

Supplementary Figure 1

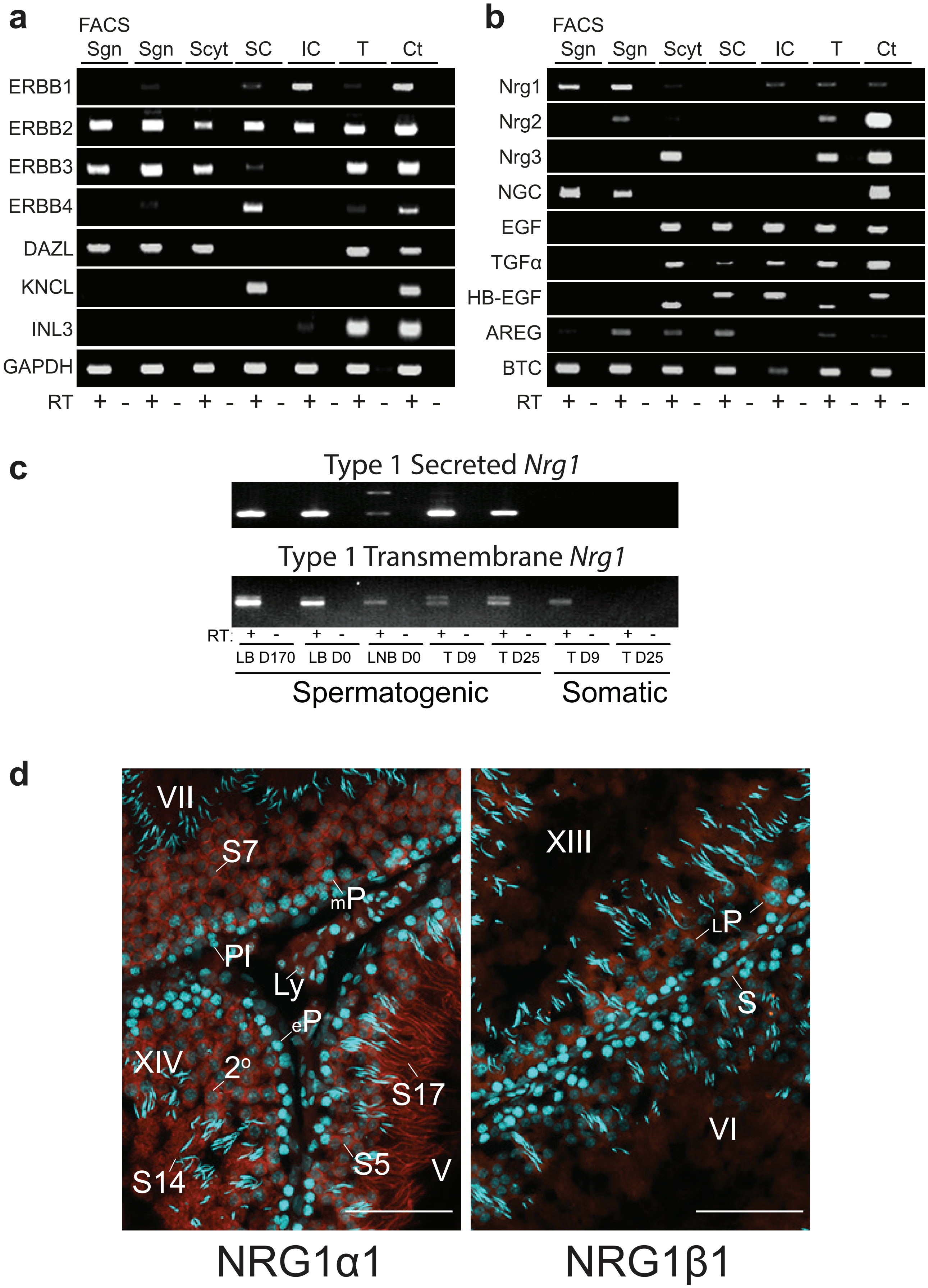

Supplementary Figure 2

a

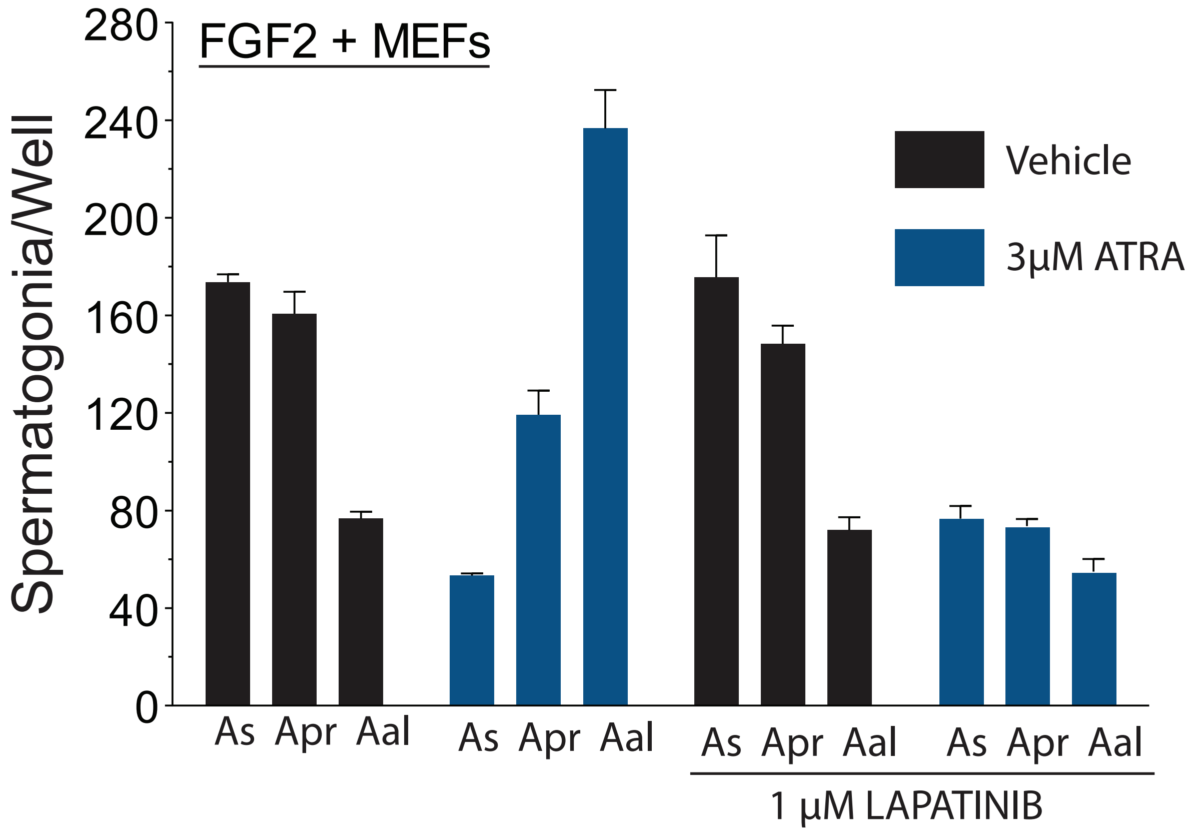

b

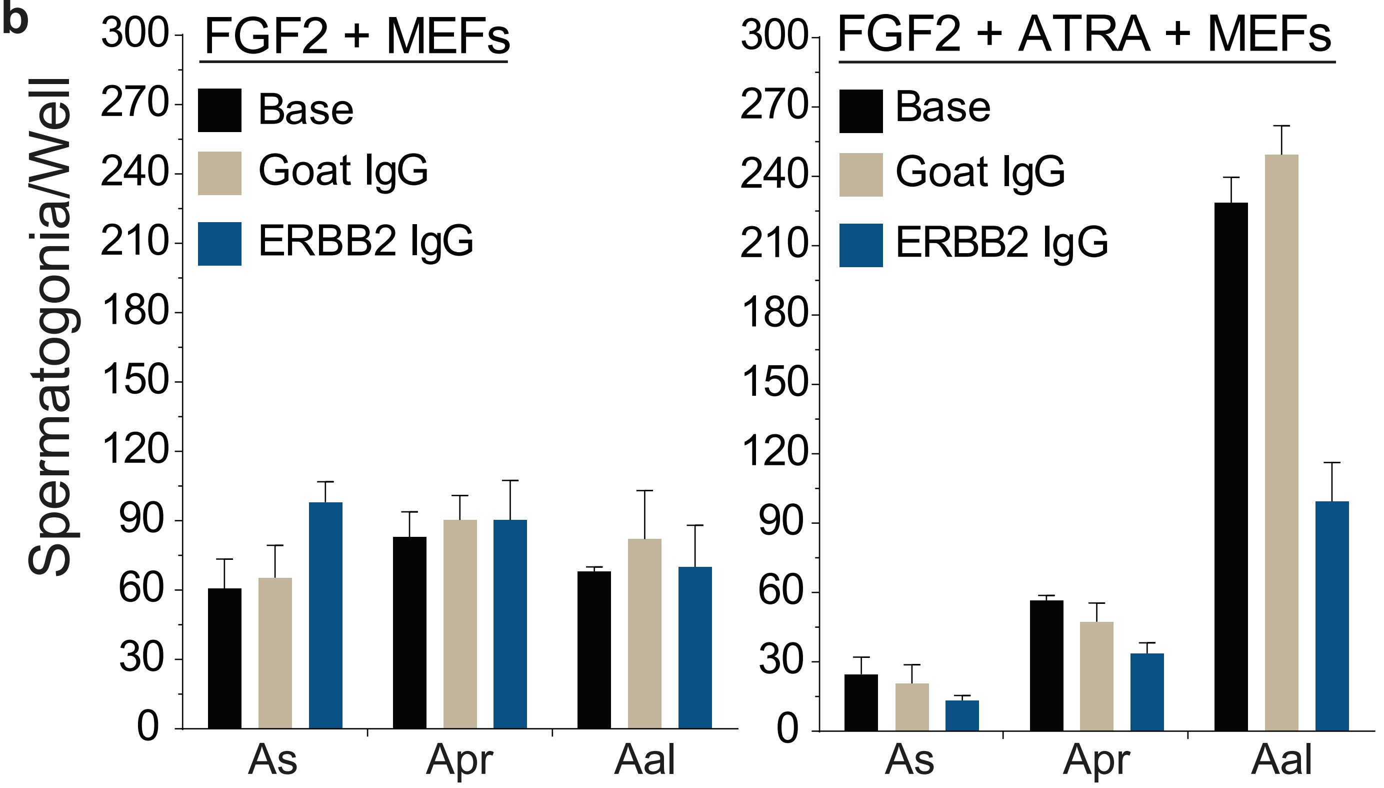

Supplementary Figure 3

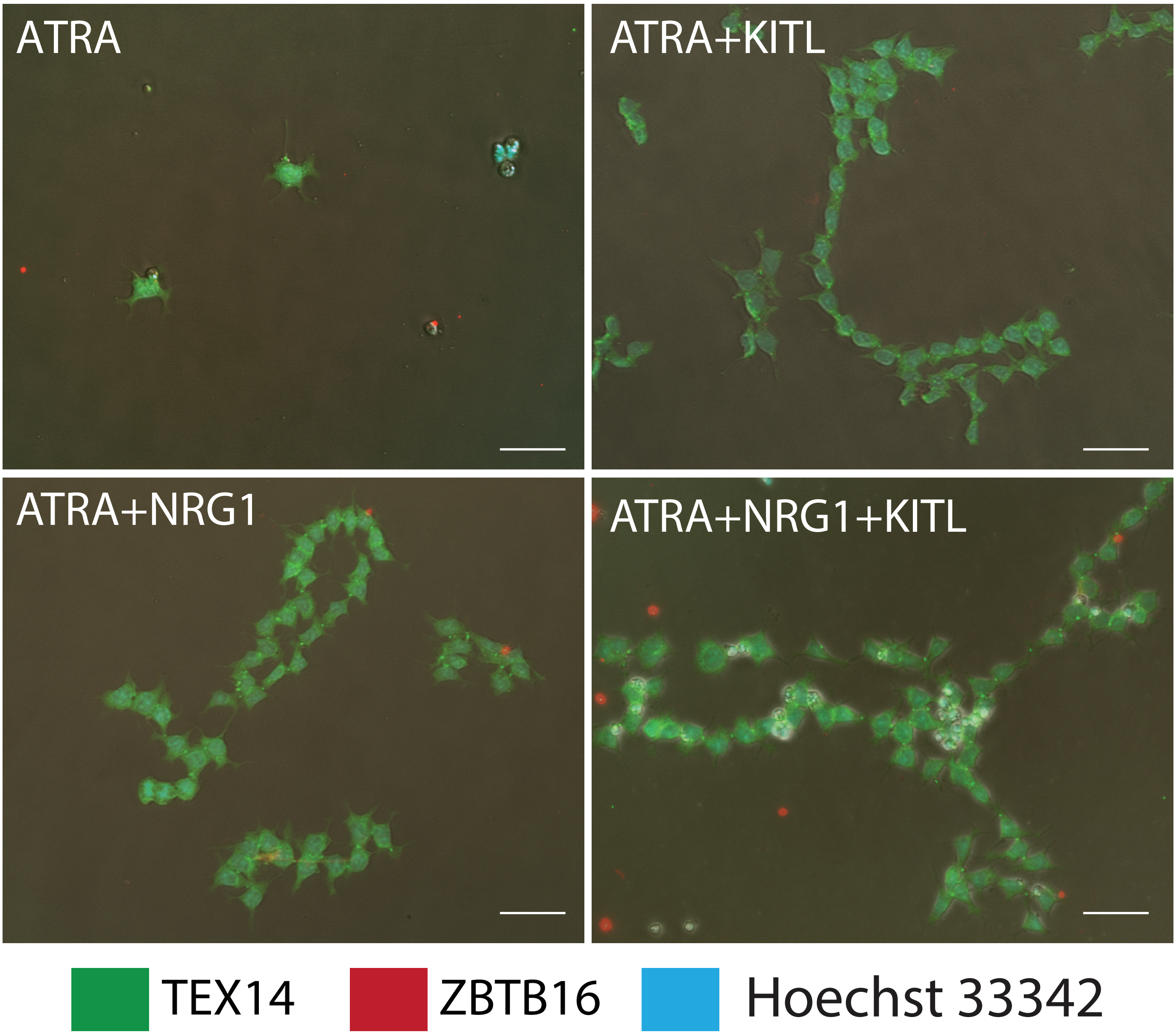

Supplementary Figure Legends

Supp Figure 1. EGF-Superfamily Ligand and Receptor Expression in Rat Testis Cells.

a. RT-PCR analysis of *Erbb*-family transcripts in testis cells isolated from D23 tg*GCS-EGFP* transgenic rats. Template cDNAs were produced from freshly isolated cultures of laminin binding type A spermatogonia (Sg), differentiating spermatogonia/early spermatocytes (Scyt), tubular somatic cells (SC), interstitial somatic cells (IC) and total testis cells (T), as described. Samples of >99.8% pure tg*GCS-EGFP*$^{+}$ spermatogonia (FACS Sgn) were isolated by flow cytometry after 170 days/14 passages in culture on mouse embryonic fibroblasts (MEFs). Controls cDNAs (+Ct) were synthesized from D23 total testes (T). RT, Reverse Transcriptase.

b. RT-PCR analysis of *Egf*-family transcripts in testis cells isolated from D23 tg*GCS-EGF*P transgenic rats. Template cDNAs were produced from freshly isolated cultures of laminin binding type A spermatogonia (Sg), differentiating spermatogonia/early spermatocytes (Scyt), tubular somatic cells (SC), interstitial somatic cells (IC) and total testis cells (T), as in panel "a". RT=Reverse Transcriptase. Controls cDNAs (+Ct) were synthesized from adult rat kidney (*Egf*), ovary (*Areg*, *Hbegf*, *Tgfaα*), brain (*Btc*, *Cspg5*, *Nrg2*) or D23 total testes (T) to identify optimal primer sets.

c. Full length Secreted and Transmembrane forms of Type-1 *Nrg1* transcripts amplified from germ cell cDNAs described in panel A using primers designed to 5-prime and 3-prime untranslated regions for variants of Type I, *Nrg1*. LB, laminin binding spermatogonia; LNB, laminin non-binding spermatogenic cells; T, EGFP^{+} Spermatogenic or EGFP^{-} Somatic testis cells flow sorted from 9 day old (D9) or 25 day old (D25) tg*GCS-EGFP* rats.

d. Immunolabeling for NRG1α1 and NRG1β1 (red cytoplasm) in adult rat testis sections. Nuclei counterstained with Hoechst 33342 dye (cyan). eP=early Pachytene Spermatocyte; mP=mid-Pachytene Spermatocyte; $_{L}$P=late Pachytene spermatocyte; S5, S8, S14, S17=Step 5, 8, 14, 17 spermatids; 2°=secondary spermatocyte; L=Leydig cells; S=Sertoli cell. Roman numerals denote spermatogenic stages[1]. Scale bar, 100 μm.

Supp Figure 2. ERBB2 Inhibitors Block Syncytial Spermatogenic Cell Growth on MEFs.

a. tg*GCS-EGFP*$^{+}$ spermatogonia scored/well (±SEM, triplicate wells) after culture on MEFs *without* GDNF, but with FGF2 (SG^{F} Medium) for 1 week, and then for 6 additional days in the

same medium containing ATRA (3 µM) and/or Lapatinib (1 µM). tg*GCS-EGFP* as germ cell marker.

b. tg*GCS-EGFP*$^+$ spermatogonia scored/well (±SEM, triplicate wells) after culture on MEFs as described in panel A, but in the absence or presence of retinoic acid (3 µM), and, with or without goat IgG (6 µg/ml) or goat ERBB2 neutralizing IgG (ERBB2 IgG) (6 µg/ml). Base, SG^F Medium. A_s = A-single spermatogonia; A_{pr} = A-paired spermatogonia; A_{al} = spermatogonial syncytia containing 4-32 cells.

Supp Figure 3. Rat Differentiating Spermatogonia Cultures in SD Medium

Spermatogonial cultures after 2 days in SG^F Medium on laminin, followed by 6 days in SD Medium minus NRG1 and KITL (ATRA), or SD Medium containing NRG1 and/or KITL. ZBTB16 IgG (red), TEX14 IgG (green), Hoechst 33342 dye (blue). Scale, 50 µm
